# Supplementary material for: High-protein diets reduce plasma pro-inflammatory cytokines following lipopolysaccharide challenge in Swiss Albino mice
Source: PLoS One. 2025 Dec 15;20(12):e0338588. doi: 10.1371/journal.pone.0338588 (PMC12704898; doi:10.1371/journal.pone.0338588)
Supplement: S1 File — (PDF) [file pone.0338588.s001.pdf]

High-protein diets reduce plasma pro-inflammatory cytokines following lipopolysaccharide challenge in Swiss Albino mice

Hellen W. Kinyi [1, 2*], Charles Drago Kato[3], Lucy Ochola[4] and Gertrude N. Kiwanuka[1]

Contents

Datasets used in the study 2

Table S1:Weight of experimental animals on day 1 and week 15 2
Table S2: Cytokine profile of mice fed on high carbohydrate low lipid (HCLL) diet 2
Table S3: Cytokine profile of mice fed on high carbohydrate low protein (HCLP) diet 3
Table S4: Cytokine profile of mice fed on high protein low carbohydrate (HPLC) diet 3
Table S5: Cytokine Profile of mice fed on high protein low lipid (HPLL) diet 4
Table S6: Cytokine Profile of mice fed on high lipid low carbohydrate (HLLC) diet 4
Table S7: Cytokine Profile of mice fed on high lipid low protein (HLLP) diets 5

S1: Datasets used in the study

Table S1:Weight of experimental animals on day 1 and week 15

Diet	HCLL		HCLP		HPLC		HPLL		HLLC		HLLP	
**Animals**	**Day 1**	**Week 15**	**Day 1**	**Week 15**	**Day 1**	**Week 15**	**Day 1**	**Week 15**	**Day 1**	**Week 15**	**Day 1**	**Week 15**
M1	22.1	31.5	23.1	30	19.5	39.3	21.8	34.8	21.5	36	19.5	31.2
M2	19.8	32.9	22.4	32.4	21.5	38.6	18.2	33.9	21.7	30.9	22.6	29.6
M3	18.1	27.6	21.6	30.7	22.8	34.1	17.9	32.4	18.1	32.4	21.9	32.3
M4	23.3	31.9	21.4	35.8	21.6	33.3	20.2	33.4	19.4	30.5	21.4	28.4
M5	21.1	32.4	19.7	34.1	20.6	32.5	18.5	32.2	20.1	32.7	21.2	27.8
M6	21.2	31.6	22.8	31.5	18	32.3	18.4	32.6	18.3	30.3	19.4	28.7
F1	19.1	26	20	29.4	18.7	37.8	20.1	28.2	20.6	35.1	20.3	26.7
F2	20.8	31.3	19.6	29.6	17.4	28.8	19.2	29.8	19.5	35.6	20	29.6
F3	18	28.3	19.3	29.1	18.6	37.3	19.3	31.5	18.2	34.3	20.2	24.5
F4	17.3	32.1	19.8	29.5	20.1	30.2	20.4	29.4	18.4	32.6	20.4	25.9
F5	17.6	27.5	18.8	32.1	19.6	29.8	20.9	28.7	17.5	30.7	20	27.8
F6	17.8	29.5	19.7	28.6	16.7	28	20.1	30.5	18.1	32.5	20.7	27
**Average**	**19.7**	**30.2**	**20.7**	**31.1**	**19.6**	**33.5**	**19.6**	**31.5**	**19.3**	**32.8**	**20.6**	**28.3**
**SEM**	**0.6**	**0.7**	**0.4**	**0.6**	**0.5**	**1.1**	**0.3**	**0.6**	**0.4**	**0.6**	**0.3**	**0.6**

Key: M-Male mice. **F**-Female mice. **HCLL**-High Carbohydrate Low Lipid, **HCLP**-High Carbohydrate Low Protein, **HPLC**-High Protein Low Carbohydrate, **HPLL**-High Protein Low Lipid, **HLLC**-High Lipid Low Carbohydrate, **HLLP**-High Carbohydrate Low Protein

Table S2: Cytokine profile of mice fed on high carbohydrate low lipid (HCLL) diet

	**TNF-α**	**TNF-α_LPS**	**IL-1β**	**IL-1β_LPS**	**IL-6**	**IL-6 LPS**	**IL-10**	**IL-10 LPS**
M1	8	141	660	1407		7040	35	37
M2	7	158	305	2019		6942	32	36
M3	4	111	439	1039		6459	33	37
F1	11	137	526	2091		6068	23	32
F2	10	160	660	1714		6989	25	39
F3	11	127	483	1566		6119	28	35
**Mean**	**8.5**	**139.0**	**512.3**	**1639.3**		**6602.8**	**29.5**	**36.1**
**SEM**	**1.2**	**7.6**	**55.7**	**160.5**		**182.2**	**1.9**	**1.0**
**Paired T-test**		**0.00000973**		**0.00150**				**0.01464**

Key: M - Male mice. **F** - Female Mice. **TNF-α** - Tumour necrosis factor alpha. **LPS -** Lipopolysaccharide challenge. **IL-1β** - Interleukin-1 beta. **IL-6** - interleukin-6. **IL-10** -interleukin-10.

Table S3: Cytokine profile of mice fed on high carbohydrate low protein (HCLP) diet

	TNF-α	TNF-α_LPS	IL-1β	IL-1β_LPS	IL-6	IL-6 LPS	IL-10	IL-10 LPS
M1	10	196	439	2222		3664	28	55
M2	13	106	593	2659		3908	28	63
M3	13	167	747	3081		4643	30	36
F1	17	229	660	2161		3544	27	45
F2	13	171	704	2417		3524	17	44
F3	12	117	550	2972		3768	32.845	42
**Mean**	**12.9**	**164.3**	**615.4**	**2585.3**		**3841.6**	**27.3**	**47.5**
**SEM**	**0.9**	**19.0**	**45.8**	**157.2**		**170.6**	**2.1**	**4.0**
**T-test**		**0.000457**		**0.000044**				**0.00735**

Key: M - Male mice. **F** - Female Mice. **TNF-α** - Tumour necrosis factor alpha. **LPS -** Lipopolysaccharide challenge. **IL-1β** - Interleukin-1 beta. **IL-6** - interleukin-6. **IL-10** -interleukin-10.

Table S4: Cytokine profile of mice fed on high protein low carbohydrate (HPLC) diet

	TNF-α	TNF-α_LPS	IL-1β	IL-1β_LPS	IL-6	IL-6 LPS	IL-10	IL-10 LPS
M1	5	73	383	1439		3571	16.6	30.1
M2	7	55	383	839		3056	11.9	29.1
M3	6	55	520	1481		2743	15.1	22.0
F1	10	68	526	1352		3786	17.8	24.2
F2	10	64	533	1139		2814	20.1	27.2
F3	5	62	455	1417		3265	18.9	33.3
**Mean**	**7.0**	**62.8**	**466.7**	**1278.1**		**3205.9**	**16.7**	**27.6**
**SEM**	**0.9**	**2.9**	**28.9**	**100.7**		**169.7**	**1.2**	**1.7**
**T-test**		**0.00001**		**0.0004**				**0.0023**

Key: M - Male mice. **F** - Female Mice. **TNF-α** - Tumour necrosis factor alpha. **LPS -** Lipopolysaccharide challenge. **IL-1β** - Interleukin-1 beta. **IL-6** - interleukin-6. **IL-10** -interleukin-10.

Table S5: Cytokine Profile of mice fed on high protein low lipid (HPLL) diet

	TNF-α	TNF-α_LPS	IL-1β	IL-1β_LPS	IL-6	IL-6 LPS	IL-10	IL-10 LPS
M1	8.7	48	573.3	1021.4		2466.1	26.2	27.4
M2	8.4	36.7	526.1	1132.6		1770.3	25.2	31.9
M3	7.1	34.3	660.1	1149.4		2118.2	23.0	33.3
F1	8.6	48.4	746.9	1596.9		2421.2	31.9	22.8
F2	6.7	20.5	439.3	625.9		2338.2	31.0	25.7
F3	7.4	42.6	593.1	1111.4		2255.1	16.2	30.4
**Mean**	**7.8**	**38.4**	**589.8**	**1106.3**		**2228.2**	**25.6**	**28.6**
**SEM**	**0.3**	**4.3**	**43.4**	**126.7**		**104.6**	**2.3**	**1.6**
**T-test**		**0.0006**		**0.0021**				**0.4538**

Key: M - Male mice. **F** - Female Mice. **TNF-α** - Tumour necrosis factor alpha. **LPS -** Lipopolysaccharide challenge. **IL-1β** - Interleukin-1 beta. **IL-6** - interleukin-6. **IL-10** -interleukin-10.

Table S6: Cytokine Profile of mice fed on high lipid low carbohydrate (HLLC) diet

	TNF-α	TNF-α_LPS	IL-1β	IL-1β_LPS	IL-6	IL-6 LPS	IL-10	IL-10 LPS
M1	9	209	573	2128		3222	35	47
M2	10	274	439	2334		3645	27	41
M3	10	276	506	1889		4316	30	42
F1	12	291	747	2945		3460	18	30
F2	8	260	305	2447		4266	34	48
F3	10	374	526	2245		4214	35	46
**Mean**	**10.0**	**280.5**	**516.2**	**2331.5**		**3853.8**	**30.1**	**42.3**
**SEM**	**0.5**	**21.9**	**59.7**	**145.5**		**192.4**	**2.7**	**2.8**
**T-test**		**0.00006**		**0.00004**				**0.000008**

Key: M - Male mice. **F** - Female Mice. **TNF-α** - Tumour necrosis factor alpha. **LPS -** Lipopolysaccharide challenge. **IL-1β** - Interleukin-1 beta. **IL-6** - interleukin-6. **IL-10** -interleukin-10.

Table S7: Cytokine Profile of mice fed on high lipid low protein (HLLP) diets

	TNF-α	TNF-α_LPS	IL-1β	IL-1β_LPS	IL-6	IL-6 LPS	IL-10	IL-10 LPS
M1	14	164	439	1786		6086	30	50
M2	17	147	573	2402		6409	30	34
M3	15	172	506	1806		5396	21	32
F1	11	114	439	1596		6030	35	44
F2	11	157	305	2461		5304	40	42
F3	11	130	372	1999		6419	31	33
**Mean**	**13.2**	**147.5**	**439.3**	**2008.1**		**5940.7**	**31.1**	**39.3**
**SEM**	**1.1**	**8.9**	**38.7**	**143.8**		**198.2**	**2.5**	**3.0**
**T-test**		**0.00002**		**0.0002**				**0.0315**

Key: M - Male mice. **F** - Female Mice. **TNF-α** - Tumour necrosis factor alpha. **LPS -** Lipopolysaccharide challenge. **IL-1β** - Interleukin-1 beta. **IL-6** - interleukin-6. **IL-10** -interleukin-10.
